# The new accounting for expected adjusted effect test (AEAE test) has higher positive predictive value than a zero-order significance test

**DOI:** 10.1186/s13104-021-05545-4

**Published:** 2021-04-07

**Authors:** Kimmo Sorjonen, Gustav Nilsonne, Bo Melin, Michael Ingre

**Affiliations:** 1 Department of Clinical Neuroscience, Karolinska Institutet, Stockholm, Sweden; 2 Department of Psychology, Stockholm University, Stockholm, Sweden; 3 Institute for Globally Distributed Open Research and Education (IGDORE), Stockholm, Sweden

**Keywords:** Accounting for expected effect, Confounding, Negative predictive value, Positive predictive value, Prior probability, Regression analysis, Reliability, Simulation

## Abstract

**Objective:**

The present simulation study aimed to assess positive predictive value (PPV) and negative predictive value (NPV) for our newly introduced Accounting for Expected Adjusted Effect test (AEAE test) and compare it to PPV and NPV for a traditional zero-order significance test.

**Results:**

The AEAE test exhibited greater PPV compared to a traditional zero-order significance test, especially with a strong true adjusted effect, low prior probability, high degree of confounding, large sample size, high reliability in the measurement of predictor X and outcome Y, and low reliability in the measurement of confounder Z. The zero-order significance test, on the other hand, exhibited higher NPV, except for some combinations of high degree of confounding and large sample size, or low reliability in the measurement of Z and high reliability in the measurement of X/Y, in which case the zero-order significance test can be completely uninformative. Taken together, the findings demonstrate desirable statistical properties for the AEAE test compared to a traditional zero-order significance test.

**Supplementary Information:**

The online version contains supplementary material available at 10.1186/s13104-021-05545-4.

## Introduction

In regression analysis, it is common to include covariates to attempt to adjust for confounding variables. However, to the extent that reliability of measurement of the confounding variable is less than perfect, there will be residual confounding due to mismeasurement [1–4]. We have recently proposed a new test, which formally incorporates the reliability of the confounding variable: the Accounting for the Expected Adjusted Effect test (AEAE test). Using simulations, we showed that traditional regression methods with adjustment for a possible confounder often suffer from a high risk for type 1 errors, especially with large sample size, low reliability in the measurement of the confounder, and high reliability in the measurement of the predictor and the outcome [5]. The AEAE test mitigates the risk of type 1 errors by calculating the expected adjusted effect (Equation (Eq. 1), where *r*_*XZ*_ = correlation between predictor and confounder, *r*_*YZ*_ = correlation between outcome and confounder, *r*^*2*^_*ZZ*_ = reliability in the measurement of the confounder) and requiring that the observed adjusted effect differs significantly from this expected adjusted effect rather than from zero.

1 $$E|{\beta }_{XY.Z}|=\frac{{r}_{XZ}{r}_{YZ}(1-{r}_{ZZ}^{2})}{{r}_{ZZ}^{2}(1-{r}_{XZ}^{2})}$$

We have previously demonstrated that the AEAE test tends to have lower power than a traditional zero-order significance test (id est (i.e.) a test that expects the adjusted effect to be zero under the null hypothesis) in situations with a weak true adjusted effect. This advantage in power for the zero-order significance test is to a high degree due to its higher risk for type 1 errors. A test that gives significant results, i.e. falls prey to type 1 errors, in for example 80% of cases when the true effect equals zero will, of course, have a power > 80% if the true effect is the tiniest bit stronger than zero. Therefore, high power cannot be seen by itself as an indication of high quality of a test, just like high sensitivity does not guarantee the usefulness of a diagnostic instrument. A similar criticism can be directed at the AEAE-test: Maybe it is good at avoiding type 1 errors (has high specificity) because it has low power to detect weak, but real, true effects. Therefore, we here investigate the predictive properties of the AEAE test.

Positive Predictive Value (PPV) and Negative Predictive Value (NPV) are measures of the probability that a positive/negative finding indicates a true positive/negative condition, respectively. When it comes to statistical significance testing, positive/negative can be exchanged by significant/non-significant, respectively. Table 1 presents how to calculate the probabilities for the four possible outcomes when conducting a statistical significance test of a specific research hypothesis H1. The prior probability represents an estimated probability that the research hypothesis is true before conducting the significance test. This value can vary from very low for highly speculative hypotheses to high for more established and cautious hypotheses. With a significant result the PPV would be calculated as *P(true positive)/(P(true positive)* + *P(false positive))* and with a non-significant result the NPV would be calculated as *P(true negative)/(P(true negative)* + *P(false negative))*.

**Table 1:** Probabilities for four possible outcomes in a statistical significance test of a research hypothesis.

An outcome; P = 1			
H1 true; P = prior		H1 false; P = 1-prior	
True positive; P = prior × power	False negative; P = prior × (1−power)	False positive; P = (1−prior) × α	True negative; P = (1−prior) × (1−α)

P: probability; H1: research hypothesis; α: risk for type 1-error

The objective of the present simulation study was to assess PPV and NPV for our newly introduced test accounting for expected adjusted effect (AEAE test) and compare it to PPV and NPV for a traditional zero-order significance test.

## Main text

### Method

The method used in this paper is largely identical to the one used in our previous study, where it is described in greater detail [5]. The simulations and analyzes were conducted with R 4.0.2 statistical software [6] employing the MASS package [7] (see Additional file 1: Figure S1). In a first set of simulations, virtual participants (*N* = 20, 100, 500, or 2500) were allotted values, drawn from random standard distributions, on true Z, true X, true Y, observed Z, observed X, and observed Y. The true degree of confounding was controlled by manipulating the correlations between true Z (the confounder) and true X (the predictor) and true Y (the outcome). We used 0.1, 0.35, 0.6, or 0.85 as the values for these correlations (same for both) and drew a value from a random uniform distribution between 0 and 1 for the true degree of adjusted effect of true X on true Y. It should be noted that this latter value stands for the size of the true adjusted effect given that there actually is an independent association. However, it is also possible that the null hypothesis is correct. The participants were allotted observed Z, X, and Y values with a population correlation of 0.89 (corresponding to reliability 0.80) with their respective true values. In a second set of simulations, 0.4, 0.6, 0.8, and 0.99 were used as values for the reliabilities in Z and X/Y, 0.5 as the correlation between true X/Y and true Z, and 500 as sample size.

We ran 1000 simulations for each of the 16 combinations of sample size and true degree of confounding (first set of simulations) and the 16 combinations of reliabilities in the measurement of Z and X/Y (second set), respectively. In each simulation, ordinary least squares regression was used to analyze the observed effect of X on Y, adjusting for Z. Logistic regression was used in each of the 2 × 16 combinations to calculate the function for the association between the true adjusted effect and the probability for a significant (*p* < 0.05) observed adjusted effect. The predicted value from this function equals the risk for type 1 error (α) when true adjusted effect = 0, and equals power when true adjusted effect > 0. For three different prior probabilities (0.1, 0.5, and 0.9), we calculated the probabilities for a true positive, a false negative, a false positive, and a true negative outcome, respectively, in accordance with the formulas in Table 1. In a last step we calculated positive predictive value (PPV) and negative predictive value (NPV) in accordance with formulas mentioned in the introduction.

The significance of the observed effect of X on Y, adjusting for Z, was calculated in two different ways. In a traditional zero-order significance test (Eq. 2), the coefficient for the adjusted effect (β_XY.Z_) is divided by its standard error (*SE*(β_XY.Z_)). This gives a *T*-value and if the corresponding *p*-value is significant (usually defined as *p* < 0.05), an independent association between X and Y adjusting for Z is assumed, i.e. it is assumed to differ from zero. In the test accounting for expected adjusted effect (AEAE test), we have, instead, the difference between the observed and the expected, as given by Eq. 1, coefficients for the adjusted effect in the numerator (Eq. 3). Here a significant *T*-value would indicate that the observed adjusted effect differs from the expected due to spurious reasons.

2 $${T}_{0}=\frac{{\beta }_{XY.Z}}{SE({\beta }_{XY.Z})}$$

3 $${T}_{AEAE}=\frac{{\beta }_{XY.Z}-E|{\beta }_{XY.Z}|}{SE({\beta }_{XY.Z})}$$

### Results and discussion

#### Positive predictive value (PPV)

**Fig. 1:**
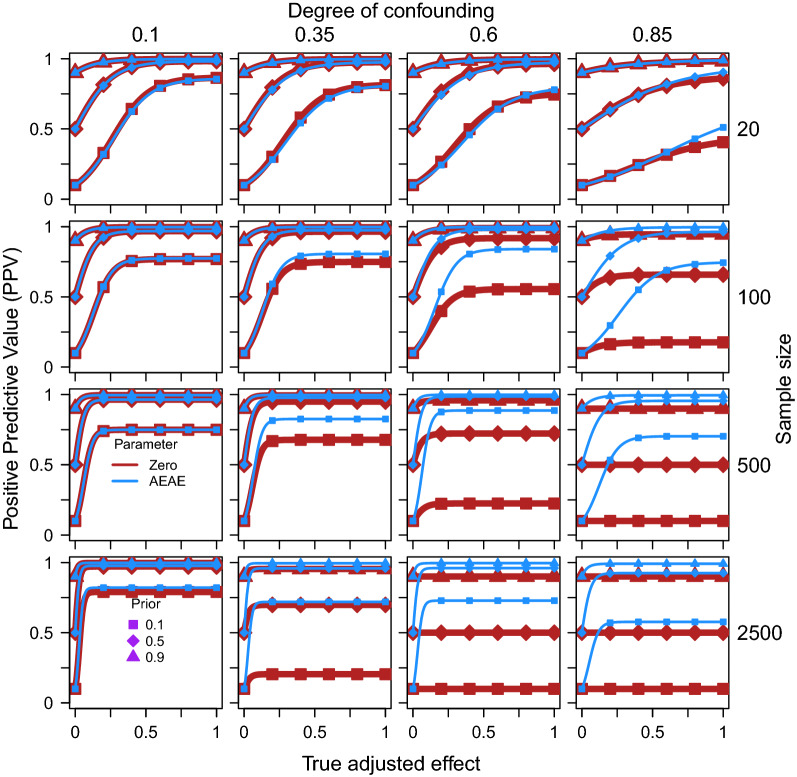
Positive predictive value (PPV) for zero-order significance tests (Eq. 2) of the effect of X on Y while adjusting for Z (thick red line) as well as when accounting for the expected adjusted effect (AEAE test, Eqs. 1, 3, blue line) as functions of the true adjusted effect, separately for three different prior probabilities (different markers), four degrees of confounding (i.e. correlation between true Z and true X/Y, columns) and four sample sizes (rows). The reliability in measurement of X/Y/Z was fixed at 0.8 in these simulations

**Fig. 2:**
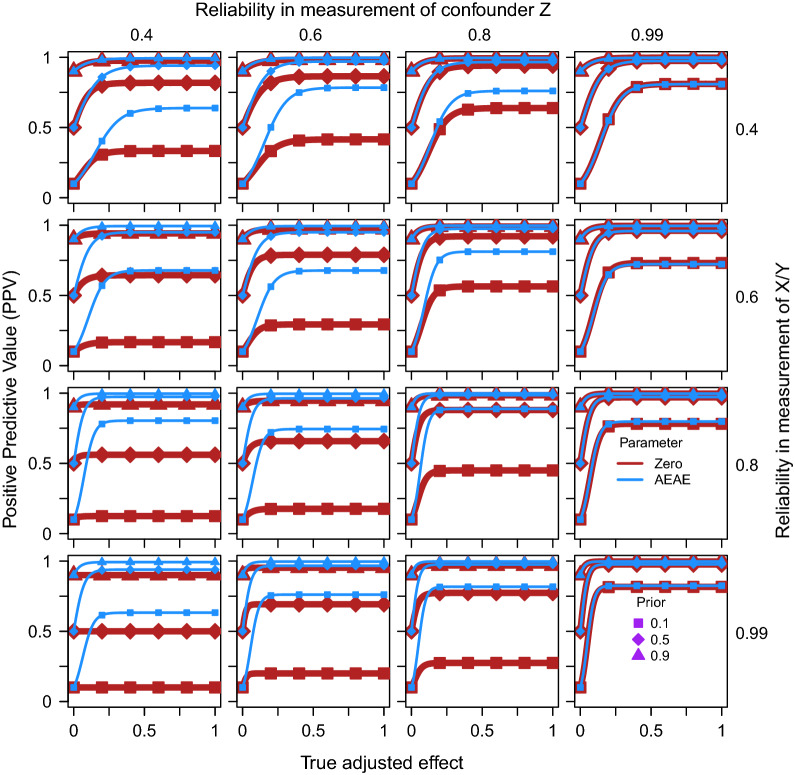
Positive predictive value (PPV) for zero-order significance tests (Eq. 2) of the effect of X on Y while adjusting for Z (thick red line) as well as when accounting for the expected adjusted effect (AEAE test, Eqs. 1, 3, blue line) as functions of the true adjusted effect, separately for three different prior probabilities (different markers), four degrees of reliability in the measurement of Z (columns) and four degrees of reliability in the measurement of X/Y (rows). The sample size was fixed at 500 and the degree of confounding at 0.5 in these simulations

Figures 1 and 2 show that the AEAE test exhibits higher PPV than the zero-order significance test. The difference increases with the size of the true adjusted effect, the degree of confounding, the sample size, and the reliability in the measurement of X/Y, while the difference is attenuated by the prior probability and the reliability in the measurement of Z. For example, in Fig. 1, with degree of confounding = 0.6, sample size = 500, prior = 0.1, and true adjusted effect (if H1 is true) = 0.4, PPV for the zero-order significance test equals 0.225 while it equals 0.887 for the AEAE test. With a high degree of confounding in combination with a large sample size (lower right panels in Fig. 1), and a low reliability in the measurement of Z in combination with a high reliability in the measurement of X/Y (lower left panel in Fig. 2), the zero-order test is completely uninformative, as it always gives significant results irrespective of the size of the true adjusted effect, and PPV is predicted to be the same as the prior. The positive effect of accounting for expected adjusted effect on PPV is not surprising, considering the decreasing effect it was demonstrated to have on the risk for type 1 error in our previous study [5].

It can be noted that the traditional zero-order significance test of an adjusted effect works best (has highest PPV) when it is least needed, i.e. with a low degree of true confounding. The AEAE test is much less sensitive to the degree of true confounding. Similarly, the PPV of the AEAE test is less sensitive to the influence of sample size and reliability in the measurements of the outcome, the predictor, and the possible confounder. Somewhat paradoxically, the PPV of the zero-order significance test decreases with an increase in sample size and the reliability in measurement of the outcome and the predictor, reflecting an increase in false positive findings. These results highlight that when the degree of confounding is moderate or high, the AEAE test can mitigate strongly against residual confounding.

#### Negative predictive value (NPV)

Although not as large as the difference in PPV, it is apparent in Additional file 2: Figure S2 and Additional file 3: Figure S3 that the zero-order significance test tends to have higher NPV compared to the AEAE test, at least with a high prior probability in combination with a high degree of confounding and a smallish sample size, or in combination with a low reliability in the measurement of the confounder Z. These conditions coincide with those indicated to give the zero-order test a power advantage compared to the AEAE test in our previous study [5]. For example, in Additional file 2: Figure S2, with degree of confounding = 0.85, sample size = 100, prior = 0.9, and true adjusted effect (if H1 is true) = 0.4, NPV for the zero-order significance test equals 0.838 while it equals 0.154 for the AEAE test. However, with a high degree of confounding in combination with a large sample size (lower right panels in Additional file 2: Figure S2), and a low reliability in the measurement of Z in combination with a high reliability in the measurement of predictor X and outcome Y (lower left panel in Additional file 3: Figure S3), the zero-order test is completely uninformative, as it always gives significant results irrespective of the size of the true adjusted effect, and the NPV cannot be calculated.

## Conclusions

The present study demonstrates a high probability that a statistically significant effect from a traditional zero-order significance test of a predictor X on an outcome Y while adjusting for a possible confounder Z is false, i.e. falling prey to type 1 error and having low positive predictive value (PPV). This finding is consistent with a body of literature demonstrating limited reproducibility in empirical sciences [8–12]. We further show that this increased risk can be mitigated by calculating the expected adjusted effect and requiring the observed adjusted effect to differ significantly from this expected effect (AEAE test) rather than interpreting directly a significant deviation from zero.

Taken together, the findings indicate desirable statistical properties for the AEAE test compared to a traditional zero-order significance test, and it is our hope that the AEAE test can contribute to alleviating the reproducibility crisis that psychology and other empirical sciences experience.

## Limitations

The presented AEAE test is, so far, only applicable to linear regression adjusting for a single confounder. However, future extension to other analyses, for example logistic and Cox regression, and to situations with more than one possible confounder, should not be impossible.

## Supplementary Information

**Additional file 1: Figure S1**. Illustration of the present simulation, with various degrees of confounding effects of true Z on true X/Y, various degrees of true adjusted effects of true X on true Y, and various degrees of reliability in the measurement of Z/X/Y. The significance of the effect of observed X on observed Y while adjusting for observed Z was calculated. Image is adapted from Sorjonen et al. (2020).

**Additional file 2: Figure S2**. Negative predictive value (NPV) for zero-order significance tests (Eq. 2) of the effect of X on Y while adjusting for Z (thick red line) as well as when accounting for the expected adjusted effect (AEAE test, Eqs. 1, 3, blue line) as functions of the true adjusted effect, separately for three different prior probabilities (different markers), four degrees of confounding (i.e. correlation between true Z and true X/Y, columns) and four sample sizes (rows). The reliability in measurement of X/Y/Z was fixed at 0.8 in these simulations.

**Additional file 3: Figure S3**. Negative predictive value (NPV) for zero-order significance tests (Eq. 2) of the effect of X on Y while adjusting for Z (thick red line) as well as when accounting for the expected adjusted effect (AEAE test, Eq. 1 and Eq. 3, blue line) as functions of the true adjusted effect, separately for three different prior probabilities (different markers), four degrees of reliability in the measurement of Z (columns) and four degrees of reliability in the measurement of X/Y (rows). The sample size was fixed at 500 and the degree of confounding at 0.5 in these simulations.

## Data Availability

The script, which also generates the data, is available at Open Science Framework at https://osf.io/js3yb/.

## Abbreviations

AEAE: Accounting for expected adjusted effect
Eq.: Equation
PPV: Positive predictive value
NPV: Negative predictive value
i.e.: Id est
SE: Standard error
