# Supplementary figures and images for: The new accounting for expected adjusted effect test (AEAE test) has higher positive predictive value than a zero-order significance test
Source: BMC Res Notes. 2021 Apr 7;14:129. doi: 10.1186/s13104-021-05545-4 (PMC8028113; doi:10.1186/s13104-021-05545-4)

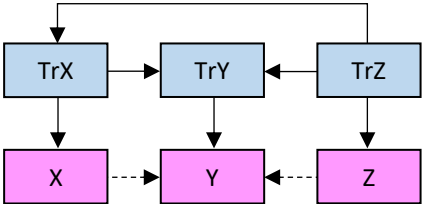

Supplement: Supplementary file 1 — Additional file 1: Figure S1. Illustration of the present simulation, with various degrees of confounding effects of true Z on true X/Y, various degrees of true adjusted effects of true X on true Y, and various degrees of reliability in the measurement of Z/X/Y. The significance of the effect of observed X on observed Y while adjusting for observed Z was calculated. Image is adapted from Sorjonen et al. (2020). [file 13104_2021_5545_MOESM1_ESM.pdf]

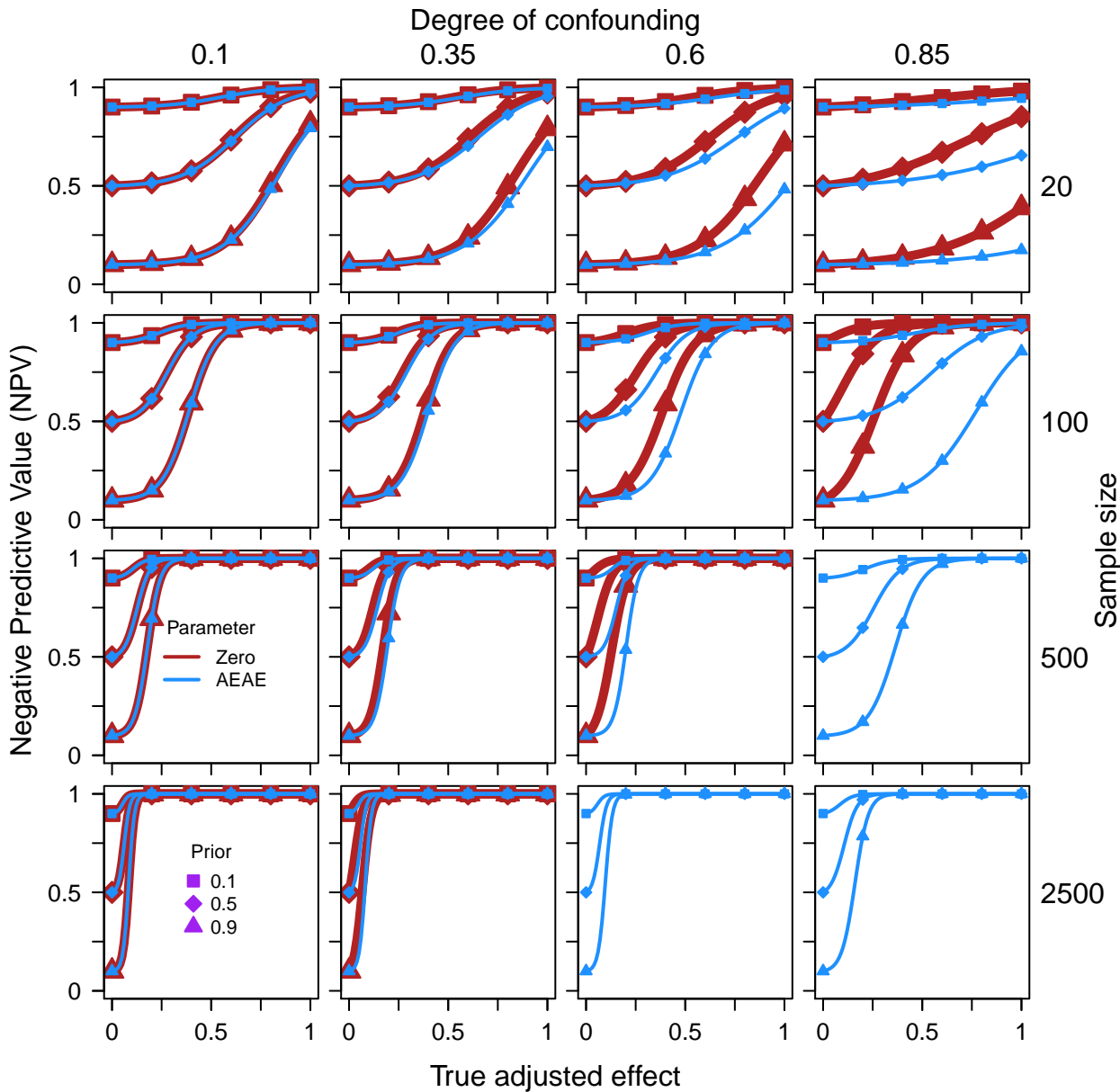

Supplement: Supplementary file 2 — Additional file 2: Figure S2. Negative predictive value (NPV) for zero-order significance tests (Eq. 2) of the effect of X on Y while adjusting for Z (thick red line) as well as when accounting for the expected adjusted effect (AEAE test, Eqs. 1, 3, blue line) as functions of the true adjusted effect, separately for three different prior probabilities (different markers), four degrees of confounding (i.e. correlation between true Z and true X/Y, columns) and four sample sizes (rows). The reliability in measurement of X/Y/Z was fixed at 0.8 in these simulations. [file 13104_2021_5545_MOESM2_ESM.pdf]

Reliability in measurement of confounder Z

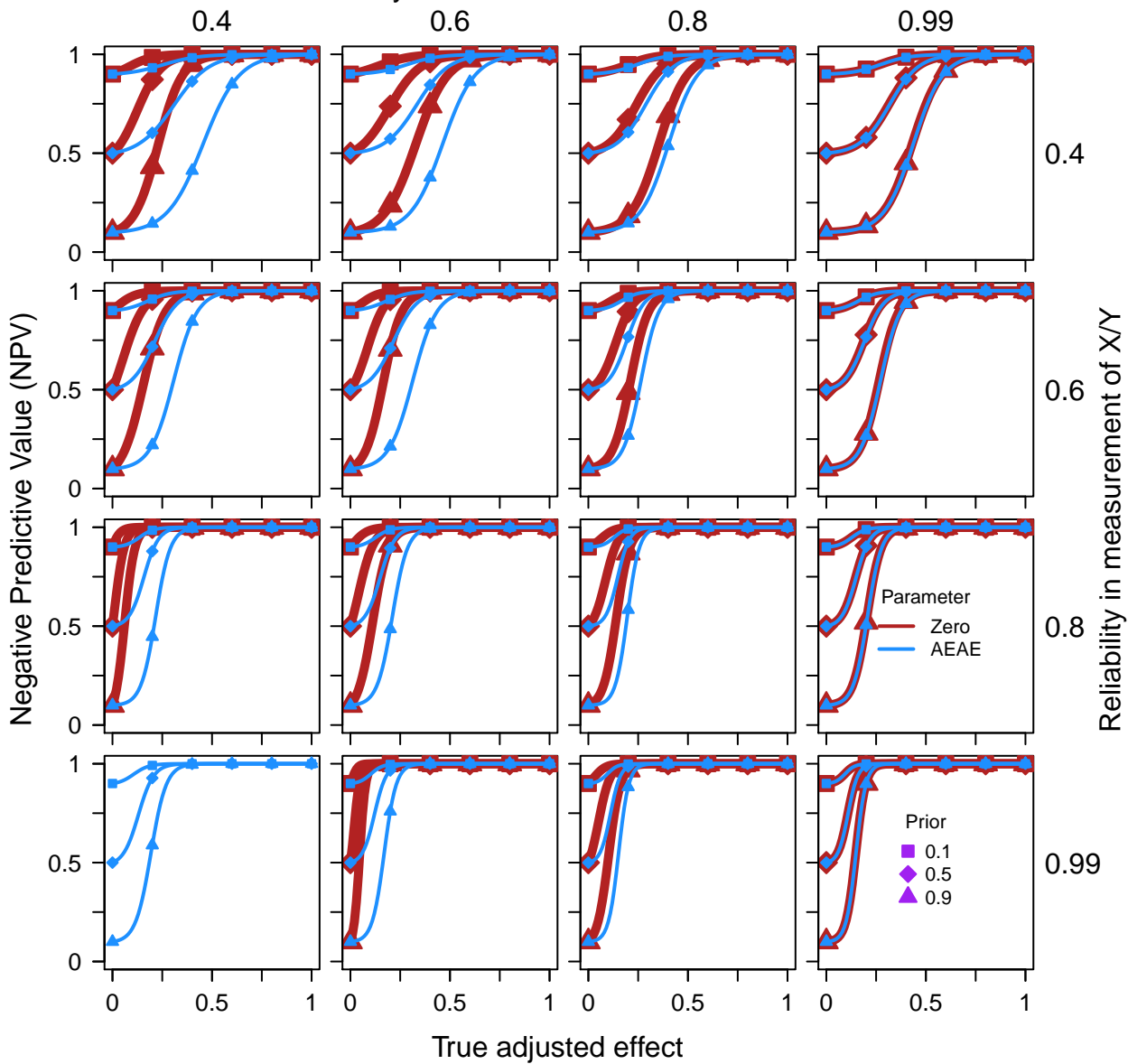

Supplement: Supplementary file 3 — Additional file 3: Figure S3. Negative predictive value (NPV) for zero-order significance tests (Eq. 2) of the effect of X on Y while adjusting for Z (thick red line) as well as when accounting for the expected adjusted effect (AEAE test, Eq. 1 and Eq. 3, blue line) as functions of the true adjusted effect, separately for three different prior probabilities (different markers), four degrees of reliability in the measurement of Z (columns) and four degrees of reliability in the measurement of X/Y (rows). The sample size was fixed at 500 and the degree of confounding at 0.5 in these simulations. [file 13104_2021_5545_MOESM3_ESM.pdf]
